# Shrub encroachment alters plant trait response to nitrogen addition in a semi-arid grassland

**DOI:** 10.3389/fpls.2023.1103371

**Published:** 2023-03-16

**Authors:** Dan Li, Yanshu Liu, Xiaohui Yang, Xiao Zhang, Zhongjie Shi

**Affiliations:** ^1^ Institute of Desertification Study, Institute of Ecological Conservation and Restoration, Chinese Academy of Forestry, Beijing, China; ^2^ Key Laboratory of Land Consolidation and Rehabilitation, Land Science and Technology Innovation Center, Land Consolidation and Rehabilitation Center, Ministry of Natural Resources, Beijing, China

**Keywords:** *Leymus chinensis*, leaf traits, growth, nitrogen addition, shrub patches

## Abstract

Encroachment of shrubs over large regions of arid and semi-arid grassland can affect grassland traits and growth under a background of increasing nitrogen (N) deposition. However, the effects of N input rates on species traits and the growth of shrubs on grasslands remain unclear. We examined the effects of six different N addition rates on the traits of *Leymus chinensis* in an Inner Mongolia grassland encroached by the leguminous shrub, *Caragana microphylla*. We randomly selected 20 healthy *L. chinensis* tillers within shrubs and 20 tillers between shrubs in each plot, measuring the plant height, number of leaves, leaf area, leaf N concentration per unit mass (LNC_mass_), and aboveground biomass. Our results showed that N addition significantly enhanced the LNC_mass_ of *L. chinensis*. The aboveground biomass, heights, LNC_mass_, leaf area, and leaf number of plants within the shrubs were higher than those between shrubs. For *L. chinensis* growing between shrubs, the LNC_mass_ and leaf area increased with N addition rates, leaf number and plant height had binomial linear relationships to N addition rates. However, the number of leaves, leaf areas and heights of plants within shrubs did not vary under various N addition rates. Structural Equation Modelling revealed N addition had an indirect effect on the leaf dry mass through the accumulation of LNC_mass_. These results indicate that the response of dominant species to N addition could be regulated by shrub encroachment and provide new insights into management of shrub encroached grassland in the context of N deposition.

## Introduction

Grasslands are the most extensively distributed ecosystems in the world (Zhao et al., 2020; Zhou et al., 2020), but woody shrub encroachment has become a problem in recent decades (Van Auken, 2009; Yan et al., 2019). Shrub encroachment of grasslands has attracted the attention of ecologists for more than a century (D’Odorico et al., 2012). The cover and density of native shrub species have increased rapidly due to multiple factors, including climate change, overgrazing, fire prevention and their interactions (Van Auken, 2009). Previous studies revealed that shrub encroachment is of benefit for nutrient cycling, the accumulation of soil organic matter, and understorey plants of grassland communities (Hibbard et al., 2001; Eldridge et al., 2011; Deng et al., 2021). Taller shrubs can accumulate more soil water and nutrients than grass (Saixiyala et al., 2017; Ward et al., 2018a), while acting as nurse species for understorey plants, avoiding intake by herbivores (Burrows et al., 1990; Dean et al., 1999; Zhou et al., 2018).

Recent remote sensing research found that woody plant encroachment could increase the leaf areas of plants and global vegetation greening, thereby improving the productivity of semi-arid grasslands (Deng et al., 2021). However, shrub patches are more adaptable than forbs and grasses as they have the capacity to obtain more resources; thus, they can outcompete them. Further, taller shrubs can intercept sunlight, thereby suppressing the growth of understorey plants by limiting their opportunities for photosynthesis (Staver et al., 2011; Liu et al., 2022). Previous researches revealed that shrub encroachment can decrease the richness and diversity of grassland communities, thereby reducing the livestock carrying capacity by lowering the productivity of palatable herbs in arid and semi-arid grasslands (Zhou, 1990; Ratajczak et al., 2012; Cai et al., 2020).

Nitrogen plays a decisive role in the healthy development of grassland communities. N fertilization is widely employed as an effective management tool for enhancing primary production, particularly in arid and semi-arid regions where nitrogen is one of main limiting factors for the growth of plants (LeBauer and Treseder, 2008; Erisman et al., 2013; Greaver et al., 2016). N inputs have been shown to alter the composition of grassland communities and their functional traits (Tatarko and Knops, 2018), and increase productivity (Tang et al., 2017; Lu et al., 2021). However, the effects of N addition on the diversity of grassland communities are uncertain at present. Some researchers observed that grassland diversity was rejuvenated with N enrichment (Isbell et al., 2013; Storkey et al., 2015), while others found that long-term N enrichment reduced grassland diversity, which led to a reduction in ecosystem functionality (Niu et al., 2018; Midolo et al., 2019). These distinct responses may be due to different reactions between individual plants and environmental conditions (Bai et al., 2015; You et al., 2017). Therefore, from the perspective of plant traits and growth, it is necessary to explore the complex mechanisms involved in their responses to N addition.

Plant traits reflect the core attributes of vegetation in response to environmental changes, which are used to explore the impacts of environmental changes on individual plants and grassland communities (Diaz et al., 2016; Stotz et al., 2021). For example, the plasticity of leaf traits is impacted by environmental changes (Ordonez et al., 2009). Specifically, earlier research from the Mediterranean indicated that the specific leaf area and leaf dry matter content might explain >90% of the variation in leaf traits across various environments (Roche et al., 2004). Study of a semi-arid grassland revealed that the stem-leaf biomass ratio is more responsive to grazing, while the specific leaf area is more sensitive to N enrichment (Zheng et al., 2022). Further investigations have indicated that the addition of N has a negative effect on the leaf dry matter content (Mao et al., 2014; Zheng et al., 2017; Sun et al., 2022). However, there is no consensus on the responses of specific leaf area, leaf P and N concentrations to N addition between different species (Huang et al., 2008; Zhang et al., 2018; Su et al., 2021). Moreover, the mechanisms of N addition effects on the leaf traits of dominant plants in shrub encroached grasslands have not received adequate attention.

The Xilin Gol grassland is a temperate Eurasian grassland in arid and semi-arid regions, in which *L. chinensis* is a dominant species with a high forage value (Liu et al., 2011). This grassland has become encroached upon by a spiny leguminous shrub, *C. microphylla*. Leguminous shrubs with spiny leaf margins are relatively unpalatable, and can increase the nutrient status of the habitat *via* nitrogen fixation (Peng et al., 2013). So, the shrubs have a greater chance of survival than herbaceous vegetation in nutrient limited grasslands. Thus, shrub expansion is likely to limit the growth of grasses and alter rangeland structures (Peng et al., 2013; Shen et al., 2016; Zhou et al., 2019). Further research revealed that appropriate N addition was beneficial for accelerating the reproductive capacity of *L. chinensis*, maintaining high-quality pastures in the Xilin Gol grassland (Liu et al., 2021), and increasing community productivity (Zheng and Ma, 2018). However, it remains unclear how the biomass and leaf traits of dominant plant species are modified under various N addition rates in leguminous shrub encroached grassland, and how the presence of shrub patches impacts the effects of N addition on dominant herb species.

The objectives of this study were to explore the effects of various N addition rates on plant height, biomass, and the leaf traits of *L. chinensi*s between shrub patches and within shrub patches. We conducted an N addition experiment in a leguminous shrub encroached grassland with six different N addition rates, ranging from 0 to 20 g N·m^-2^·yr^-1^. We aimed to answer two questions: (1) Does N addition influence the biomass and leaf traits of *L. chinensis* growing between and within shrubs? (2) Were the effects of nitrogen addition on the leaf traits of *L. chinensis* regulated by shrubs?

## Materials and methods

### Study site

**Figure 1:**
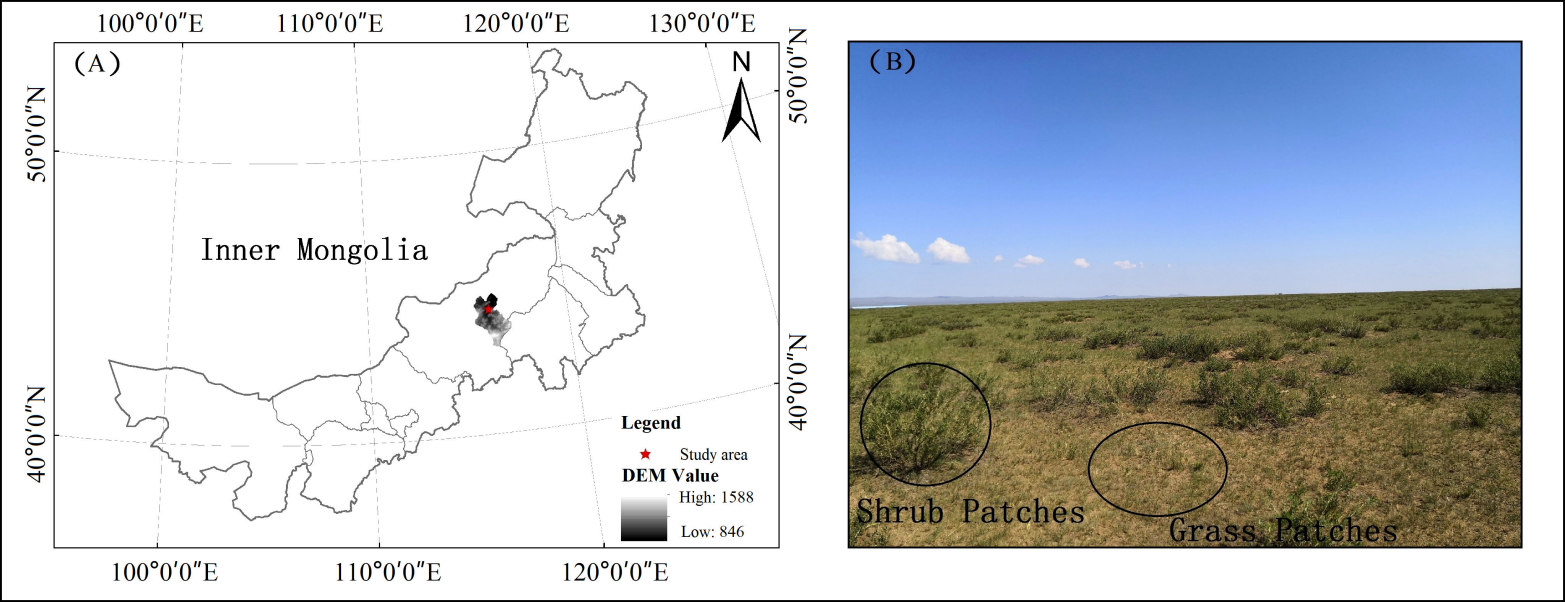
**(A)** Location of the study area in the Xilin Gol grassland of Inner Mongolia, China. **(B)** Detailed image of the shrub patches and grass patches in shrub encroached grassland.

This experiment was conducted on a semiarid grassland of Inner Mongolia, China (44°18′ - 44°25′N, 116°03′ - 116°07′E), belonging to the Eurasian grassland. It is subject to a semi-arid continental climate (Liu et al., 2019) (**Figure 1**). Based on prolonged meteorological data (1988-2018), the mean annual temperature (MAT) and mean annual precipitation (MAP) were 3.37°C and 273.21 mm, respectively. In 2019, the precipitation was 176.80 mm with 75% falling during the growing season, and the temperature was 4.33°C. The soil is a calcareous chestnut soil (Calcic Chernozem) according to the IUSS Working Group WRB (2006) and the slope is <1%. A perennial rhizomatous grass (*L. chinensis*) is the dominant species in the community, and the co-occurring species included *Cleistogenes squarrosa*, *Lespedeza daurica*, *emarrhena asphodeloides*, etc. (Liu et al., 2011). The leguminous shrub, *C. microphylla*, is invading the study area (Peng et al., 2013). Prior to the experiment, the site had no fertilizers applied.

### Experimental design

We established the N addition field experiment in 2018, following a randomized complete block design. Before the onset of the experiment, the site was moderately grazed. A set of six N addition rates were employed, including a control (0) and 2.5, 5, 10, 15, and 20 g N·m^-2^·yr^-1^, denoted as N0, N2.5, N5, N10, N15, and N20, respectively. Each treatment had four replicates, a total of 24 plots. Each plot was 20 m × 50 m, separated by 5 m walkways. The selected shrub patches were nested within the N addition rates, where four shrub patches and four grass patches were selected for the collection of individual samples in each plot, and marked as within shrub patches or between shrub patches according to position. Grass patches categorized as between shrubs were >1 m from shrubs to minimize the influence of neighboring shrubs. Urea (CO(NH_2_)_2_) was applied in early June each year immediately prior to rainfall.

### Field sampling and measurements

During July 2019, 40 tillers were selected from each plot, 20 from shrub patches and 20 from grass patches between shrubs. Following height measurements, the samples were cut at ground level, placed into sealed bags which were stored in a mobile refrigerator to keep fresh, and then transferred to the laboratory to measure leaf trait parameters. Each tiller was separated as stem and leaves, and the number of leaves was recorded. The second whole and healthy leaf from the top of each tiller was selected for width and length measurement using a ruler. The leaf length was recorded as the maximum value along the midrib, whereas the leaf width was the maximum width perpendicular to the midrib. If the second leaf was incomplete, the closest whole leaf was selected. The leaf area was measured with a leaf area meter (Li -3100, Li-Cor, Lincoln, USA). Subsequently, the samples were oven-dried at 65°C for 48 h to estimate the dry mass. Finally, the leaf length width ratio was calculated along with the specific leaf area, aboveground biomass, and stem-leaf biomass ratio (Zheng et al., 2022).

The leaf samples were ground into powder using a ball mill (Retsch MM 400; Retsch, Haan, Germany) before analysis of nutrient concentrations. The leaf N concentration per unit mass (LNC_mass_, g·kg^-1^) was measured using the Kjeldahl Nitrogen Determination method. The leaf N concentration per unit area (LNC_area_) (Equation 1) and leaf N absorption per tiller (leaf N absorption) (Equation 2) were then calculated.


(1) $$LNC_{area}=\frac{LNC_{mass}}{Specific\;leaf\;area}$$

(2) $$Leaf\;N\;absorption\;per\;tiller=LNC_{mass}\times leaf\;dry\;mass$$

### Statistical analyses

Mixed-effects models were employed to detect the individual and combined effects of N addition, as well as the presence of shrub patches on the biomass indices (leaf dry mass per tiller, biomass per tiller, and stem-leaf biomass ratio), nutrient trait parameters (LNC_mass_, LNC_area_, and leaf N absorption), and morphological trait indices (leaf length, leaf width, leaf area, number of leaves, specific leaf area, and height) of *L. chinensis* using the *lme* function from the *nlme* library (R i386 4.1.1). The tests included ‘N addition’ and ‘shrub patches’ fixed effects and ‘replication’ as a random effect. The Duncan Multiple-Range Test, Simple Linear Analysis, and Binomial Regression Analysis were used to comparatively analyze the influences of N addition rates on *L. chinensis* leaf traits between and within shrub patches. Pearson Correlation Analysis was employed to test the correlations between various plant traits using the *corrplot* package.

The coefficient of variation (CV, %) (Equation 3) was utilized to demonstrate the variation of the parameters under the different N addition rates in *L. chinensis* traits between and within shrub patches.


(3) $$CV=\frac{Standard\;deviation}{Mean}\times 100$$

Structural Equation Modelling (SEM) was performed to explore the direct and indirect effects of N addition and the presence of shrub patches on leaf dry mass per tiller. Goodness-of-fit test values were used to estimate the probability of the observed data given the model structure, such as the goodness-of-fit index (GFI), the normed fit index (NFI) and the root mean square error of approximation (RMSEA). The strength and sign of relationships between the parameters were represented by path coefficients. All statistical analyses were performed with R i386 4.1.1 and SPSS software (AMOS24 for windows, SPSS Inc., Chicago, IL, USA).

## Results

### Effects of N addition and shrub encroachment on the traits of *L. chinensis*

**Figure 2:**
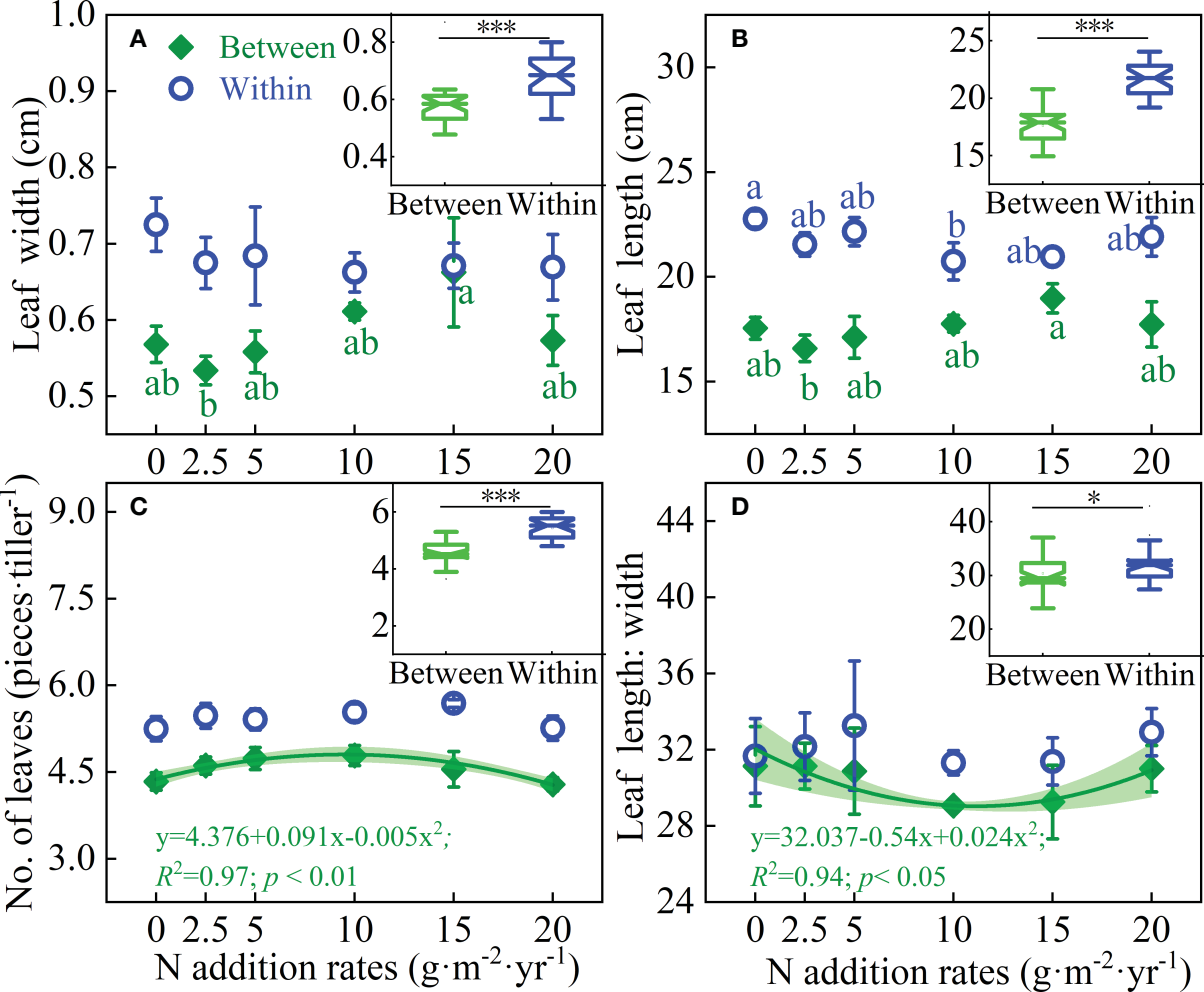
Effects of N addition rates on the **(A)** Leaf width, **(B)** Leaf length, **(C)** Number of leaves per tiller and **(D)** Leaf length width ratio of L. chinensis between and within shrub patches, and the responses of these traits to shrub patches in the inset boxplots. Different letters indicate significant differences between N addition rates at the 0.05 level. *** and * in the inset boxplots indicate significant differences in these traits between and within shrubs at the 0.001 and 0.05 levels, respectively.

Shrub encroachment significantly altered the leaf length, leaf width, leaf length width ratio, and the number of leaves of *L. chinensis* (*P* < 0.05); however, the addition of N did not affect these leaf traits. There was an interaction between N addition and shrub encroachment on leaf length (*P* < 0.05) (**Table 1**). Between shrub patches, N addition rates has no effect on leaf width and leaf length (**Figures 2A, B**). Binomial linear relationships were observed between leaf number, leaf length-width ratio and N addition rates (**Figures 2C, D**). Within shrub patches, the addition of N had no effect on leaf width, leaf number and leaf length-width ratio (**Figure 2**). The leaf length, leaf width, leaf number, leaf length-width ratio of *L. chinensis* within shrub patches increased by 23.0%, 16.6%, 20.4% and 5.6% respectively, compared with those between shrubs (**Figure 2** Boxplot).

**Table 1:** Effects of N addition and shrub patches on the traits of *L.chinensis* in a shrub encroached grassland.

	N addition		Shrub patches		Shrub patches × N addition	
	*F*	*P*	*F*	*P*	*F*	*P*
Leaf Length	0.447	0.810	201.434	**<0.001**	3.502	**0.022**
Leaf Width	0.447	0.810	35.776	**<0.001**	2.073	0.116
Number of Leaves	1.502	0.238	74.379	**<0.001**	0.427	0.824
Leaf Length-width Ratio	0.271	0.923	6.305	**0.021**	0.205	0.956
LNC_mass_	4.249	**0.010**	67.163	**<0.001**	1.004	0.444
LNC_area_	0.468	0.795	17.740	**<0.001**	1.25	0.327
Leaf N Absorption	1.928	0.139	52.335	**<0.001**	0.601	0.700
Height	0.568	0.711	518.071	**<0.001**	0.695	0.634
Leaf Dry Mass per Tiller	1.068	0.410	28.588	**<0.001**	0.664	0.656
Aboveground Biomass	0.759	0.591	44.495	**<0.001**	0.719	0.618
Stem-Leaf Biomass Ratio	0.443	0.813	27.836	**<0.001**	1.012	0.439
Leaf Area per Tiller	0.886	0.511	118.783	**<0.001**	0.663	0.656
Specific Leaf Area	2.548	0.065	33.823	**<0.001**	1.105	0.392

Bold values indicate significant impact of N addition and shrub patches on plant traits.

**Figure 3:**
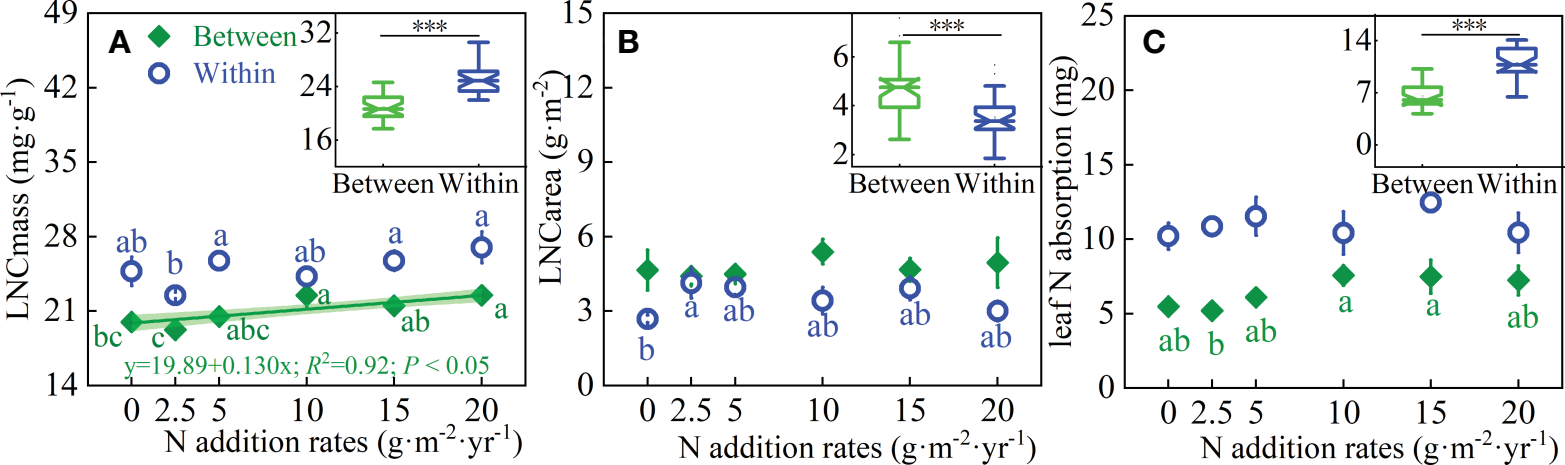
Effects of N addition rates on **(A)** LNC_mass_, **(B)** LNC_area_ and **(C)** Leaf N absorption of *L. chinensis* between and within shrub patches, and the responses of the leaf N concentrations to shrub patches in the inset boxplots. Different letters indicate significant differences between N addition rates at the 0.05 level. *** in the inset boxplots indicate significant differences in these traits between and within shrubs at the 0.001 levels, respectively.

N addition significantly affected the LNC_mass_ (*P* < 0.05) and shrub patches significant influenced LNC_mass_, LNC_area_, and leaf N absorption for *L. chinensis* (*P <*0.001); however, there were no interactions between N addition and shrub patches for these traits (**Table 1**). Between shrub patches, plant LNC_mass_ was significantly, positively correlated with N addition rate. Within shrub patches, N addition rates had a significant effect on LNC_mass_ and LNC_area_, but had no effect on leaf N absorption (**Figure 3**). The LNC_mass_ and leaf N absorption of plants within the shrub patches increased by 48.5% and 68.6%, but LNC_area_ decreased by 25.5% compared with that between shrubs (**Figure 3** Boxplot).

**Figure 4:**
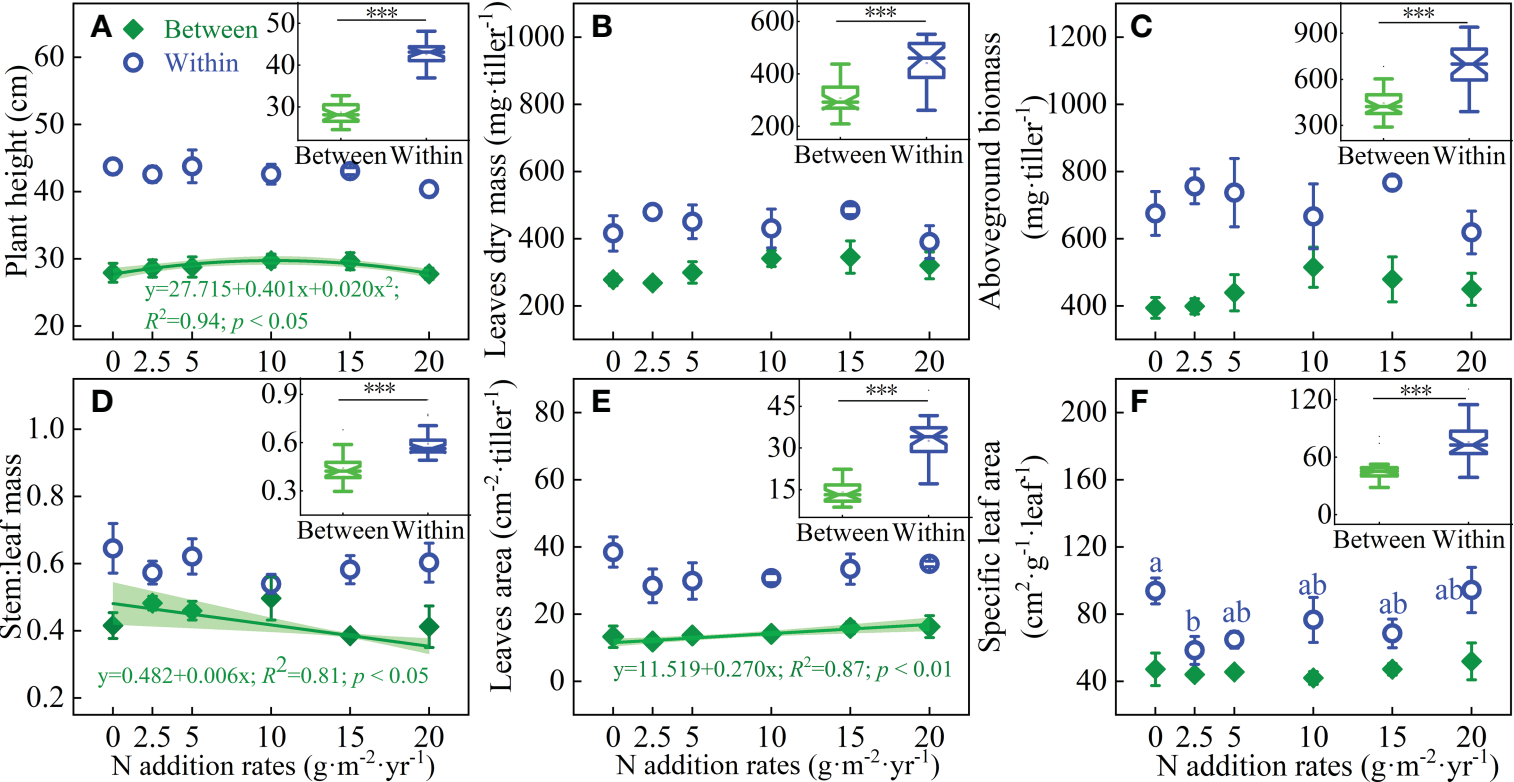
Effects of N addition on the **(A)** Plant height, **(B)** Leaf dry mass, **(C)** Aboveground biomass, **(D)** Stem leaf biomass ratio, **(E)** Leaf area and **(F)** Specific leaf area of *L. chinensis* between and within the shrub patches, and the responses of the biomass and height to shrub patches in the inset boxplots. Different letters indicate significant differences between N addition rates at the 0.05 level. *** in the inset boxplots indicate significant differences in these traits between and within shrubs at the 0.001 level.

Shrub patches significantly influenced height, leaf dry mass per tiller, aboveground biomass, stem-leaf biomass ratio, leaf area per tiller, and specific leaf area of *L. chinensis* (*P* < 0.001). However, the addition of N and the interactions between N addition and shrub patches had no impacts on these traits (**Table 1**). For the grass patches between shrubs, there was a binomial linear relationship between the height of *L. chinensis* and N addition rate (**Figure 4A**). There was negative single linear relationship between the stem-leaf biomass ratio and N addition rates (**Figure 4D**), albeit a positive single linear relationship between the N addition rates and the leaf area per tiller (**Figure 4E**). Within shrub patches, N addition had significant effects the specific leaf area of *L. chinensis* (**Figure 4F**). Compared with the growth parameters between shrubs, the height, leaf dry mass per tiller, aboveground biomass per tiller, stem-leaf biomass ratio and specific leaf area of *L. chinensis* within the shrub patches significantly increased by 48.5%, 43.1%, 57.7%, 34.1% and 64.3%, respectively. And the leaf area within shrubs is more than 1 times that between shrubs (**Figure 4** Boxplot).

at the 0.001 level.

### Relationships between the traits of *L. chinensis*

**Figure 5:**
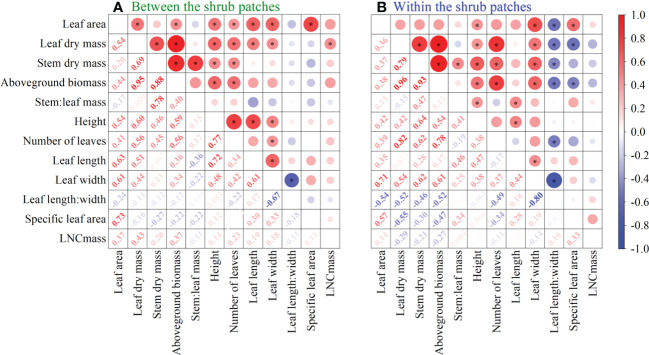
Pearson correlation analyses for plant traits of L. chinensis **(A)** Between shrub patches and **(B)** Within shrub patches. Red indicates a positive correlation, blue indicates a negative correlation, where the larger the circle area the stronger the correlation; * refers to a significant correlation at the 0.05 level.

For *L. chinensis* plants living between shrubs, the aboveground biomass was significantly positively correlated with leaf area but not with the leaf length width ratio (*P <*0.05). However, the aboveground biomass of *L. chinensis* within the shrub patches was negatively correlated with the leaf length width ratio (*P <*0.05). The aboveground biomass was significantly positively correlated with stem dry mass, plant height, and the number of leaves between or within shrub patches (*P <*0.05). Height was positively correlated with leaf length, leaf area, leaf dry mass, and aboveground biomass both between and within the shrub patches (*P <*0.05) (**Figure 5**).

### Effects of N addition on the coefficient of variation (CV) of *L. chinensis* between and within shrubs

The CV values for *L. chinensis* plants between shrubs for leaf dry mass, aboveground biomass, stem-leaf biomass ratio, leaf length, leaf width, leaf area, and LNC_mass_ were higher than those within shrub patches, whereas the CV of the LNC_area_ was lower than that within shrub patches. The CVs of the leaf dry mass and aboveground biomass in the grass patches between shrubs were increased by 25.07% and 25.36%, respectively (**Table 2**).

**Table 2:** Mean and CVs of traits for *L. chinensis* between and within shrub patches.

	Between shrub patches		Within shrub patches	
	mean	CV (%)	mean	CV(%)
Leaf Area per Tiller	14.19	10.78	32.63	10.40
Leaf Length	17.63	4.14	21.68	3.19
Leaf Width	0.58	7.15	0.68	3.03
LNC_mass_	21.04	5.84	25.00	5.66
LNC_area_	479.22	7.40	352.64	15.11
Leaf Dry Mass	308.88	9.53	441.88	7.62
Aboveground Biomass	446.02	9.54	703.33	7.61
Stem Leaf Biomass Ratio	0.44	9.08	0.59	5.75

### Structural equation models describing the effects of N addition and shrub patches on parameters

**Figure 6:**
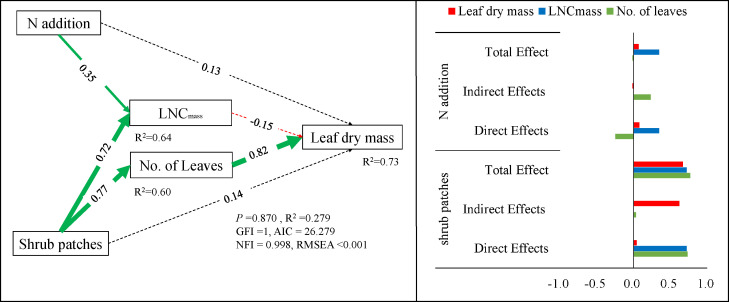
Structural equation models describing the effects of N addition and shrub patches on plant traits and leaf dry mass of *L. chinensis* in the shrub encroached grassland. The numbers adjacent to arrows (path coefficients), which are analogous to partial correlation coefficients, indicate the effect size of the relationship and may be positive (green) or negative (red). The solid line means significant effect and the dotted line means not significant effect.

We observed the full impacts of N addition and shrub patches on leaf dry mass (path =0.67 and 0.08 for the shrub patches and N addition treatment, respectively), which included the direct effects (path = 0.15 and 0.14 for the shrub patches and N addition, respectively) and indirect effects (indirect effects = 0.52 for the shrub patches; indirect effects = -0.05 for the N addition). The addition of N had indirect negative effects on the leaf dry mass through the accumulation of LNC_mass_. The presence of shrub patches had indirect positive effect on the leaf dry mass by increasing the number of plant leaves (**Figure 6**).

## Discussion

### Effects of N addition on the traits and biomass of *L. chinensis*

Nitrogen is one of the limiting factors for plant growth in semi-arid temperate grassland. Addition of N increases leaf area and N concentration, which further improves the photosynthetic and water-use efficiencies of *L. chinensis*, enhancing its competitiveness in semi-arid grasslands (Bai et al., 2008; Zheng and Ma, 2018; Tang et al., 2021). Our results indicated that the addition of N had a significant impact on the LNC_mass_ of *L. chinensis*. Lü and Han (2010) also found that N addition improved the leaf N concentrations of dominant species in a typical grassland of Inner Mongolia, China. Plants can directly absorb and utilize exogenous nitrogen added to the soil to promote organ building and metabolic transport.

Leaf traits greatly influence the acquisition of necessary resources, growth, and survival of plants (Vile et al., 2005; Liu et al., 2010; Meng et al., 2014). The leaf area and N concentrations varied between the different N treatments, with a significant increase in the leaf N concentrations under higher N rates. However, the N addition rates had no, or even negative effects, on the specific leaf area, aligning with previous field research and recent meta-analyses (Lü et al., 2016; Zhang et al., 2018; Shi et al., 2020). Our results revealed that the leaf area, LNC_mass_, and N absorption between shrub patches were stimulated by N, whereas N had no effect on the specific leaf areas and LNC_area_ between the shrubs. Further, we observed that N addition had significant non-linear effects on LNC_mass_ and LNC_area_, but not specific leaf area of *L. chinensis* within the shrub patches. These results implied that N addition affected the LNC_mass_ of plants between the shrubs; however, the responses of specific leaf area and LNC_area_ within and between the shrubs differed between N addition rates. This may have been due to the LNC_mass_ of plants between shrubs being determined primarily by soil fertility rather than shrub patches (Su et al., 2021; Sun et al., 2022). The specific leaf areas and LNC_area_ can also be affected by the local climate, soil nutrients, and plant-to-plant interactions, and may be influenced through the establishment of shrubs, particularly leguminous species, which can increase the availability of nutrients in the understory *via* nitrogen fixation (Vitousek et al., 2013; Gao et al., 2021).

The addition of N promotes plant growth and increases the height and aboveground biomass of plants in N limited environments (Bai et al., 2009; Avolio et al., 2014; Tatarko and Knops, 2018). However, once nitrogen is no longer the limiting factor, aboveground light competition gradually emerges as the key mechanism for plant height and leaf number between and within the shrubs (Borer et al., 2014). In our study, plant height and the number of leaves per tiller showed a unimodal response to N rates. Our results were supported by earlier greenhouse studies, which showed that N addition affected *L. chinensis* growth with a threshold (Liu et al., 2020). Simultaneously, the dry mass of leaves and aboveground biomass between and within the shrub patches showed no differences. Biomass and leaf area are correlated with net primary productivity and photosynthesis, where plants have some resistance to external environment changes. The effects of N addition require time to generate significant changes (Xue et al., 2019). In addition, the presence of *C. microphylla* may attenuate the facilitation of N addition on plant leaf shape and biomass (Vitousek et al., 2013).

### Positive effects of shrub encroachment on the traits of *L. chinensis*

With the continuous expansion of shrubs in grassland communities, the light, water, and nutrients available to plants in grassland ecosystems might be altered through the impacts of shrubs on soil nutrients and water, which ultimately affect the traits of these plants (Ratajczak et al., 2012; Peng et al., 2013). For example grass within legumes communities grow faster and have greater nutritive value than in communities without legumes (Gomes da Silva et al., 2021). Deng et al. (2021) and Wang et al. (2018) observed that shrub invaded areas exhibited greater grassland productivity than non-invaded areas by improving leaf area. Our research found that plant height, leaf area, leaf biomass, and LNC_mass_ of *L. chinensis* between the shrubs were significantly lower than those within shrub patches, supporting the observations that leguminous shrubs can promotes understory grass productivity. Cruz (1997) also found the N concentration of grass within shrubs was higher than between shrubs, and that the shade from shrub legumes had a positive effect on grass when N and water were limited.

The mechanisms underlying legume shrub effects on plant traits may relate to the inherent attributes of shrub species and grasses. *Leguminosae* shrubs improved soil moisture for plants and taller shrubs can accumulate more resources (such as organic matter from leaves) within shrub patches. Shrubs can also provide a greater abundance of water and nutrients for other plants within a patch, in contrast to plants between patches (Eldridge et al., 2011; Saixiyala et al., 2017; Ward et al., 2018a). Further, the shade from the shrubs reduces extreme temperatures and reduces soil evaporation, and other species within the shrub patch may be able to adapt to an understory environment through increased height growth for example. (Watt et al., 2003; Farzam and Ejtehadi, 2017).

### Interactive effects of N addition and shrub encroachment on the traits and biomass of *L. chinensis*

This study found that the responses of plant traits to N addition rates were distinct between and within shrub patches. Between the shrubs, there were linear relationships between height, number of leaves per tiller, leaf area and LNC_mass_ of plants and N addition rates. However, traits within the shrub patches showed no responses to N addition. The effects of *C. microphylla* on *L. chinensis* will be driven through plant-plant interactions (Brooker, 2006). For example, shrubs might limit the growth of *C. microphylla* plants through a reduction in radiation, and below ground competition with the shrub root zone (Fernández and Altesor, 2019). Further, leguminous shrubs might ameliorate the habitats of understorey species (Yang et al., 2017; Ward et al., 2018b).

Greater intraspecific trait variability denotes higher niche complementarity within plant communities which are beneficial to the productivity and stability of ecosystems (Bolnick et al., 2011; Cadotte, 2017). The SEM model in this study revealed that N addition increased LNC_mass_ and had indirect negative effects on the leaf dry mass, this was consistent with earlier research where N enrichment increased the variability of intraspecific trait (Fajardo and Siefert, 2018). However, we also found shrub encroachment indirectly enhanced leaf dry mass by increasing leaf number and LNC_mass_, indicating that there was tradeoff between the effect of N addition and shrub encroachment on leaf traits and biomass of *L. chinensis*. This may be because N addition can directly and efficiently increase leaf N concentration (Ren et al., 2011; Du, 2017), and Legume can also increase N availability to neighboring plants through nitrogen fixation (Peng et al., 2013). The shading of the taller shrubs might affect the adaptation of grasses to N addition; understory plants may give priority to investing resources in the aboveground parts to ensure its own survival in light-restricted habitat (Poorter et al., 2012).

## Conclusion

In the context of shrub encroachment and increasing N deposition, our research demonstrated that the aboveground biomass, heights, LNC_mass_, leaf area, and leaf number of *C. microphylla* plants within the shrub patches were higher than those between shrubs. The LNC_mass_, leaf area, leaf number and height of plant between shrubs had linear relationship with N addition rates but there was no relationship between N addition rates in these traits within the shrub patches. Concurrently, the mechanisms by which N addition regulated grass biomass were also affected by shrub patches. These results indicated that shrub encroachment might affect the responses of dominant grass traits to N deposition. Encroached shrubs reduced the sensitivity of understory grass to N deposition. Our findings have important implications in terms of maintaining the productivity of higher quality perennial grasses in grassland, and may shed light on the effects of shrub encroachment on grasslands in arid and semi-arid grassland. Therefore, in the context of N deposition, management of shrub encroached grassland must consider the effects of shrubs.

## Data availability statement

The raw data supporting the conclusions of this article will be made available by the authors, without undue reservation.

## Author contributions

Conceptualization, DL, YL, and XY; Methodology, DL and XZ; Writing original draft, DL and YL; Writing-review and editing, YL, XY, and ZS; Funding acquisition, XY and YL. All authors contributed to the article and approved the submitted version.
